# Interspecific variation of functional traits in saplings of three Amazonian species under drought stress and recovery

**DOI:** 10.1093/aobpla/plaf073

**Published:** 2026-01-08

**Authors:** Zilza T M Guimarães, Debora Coelho-Silva, José C R Soares, Guilherme S Modolo, Alaíde de O Carvalho, André H B Neves, Thalita V M S Fernandes, Daniel M Johnson, Daniel Markewitz, Marciel J Ferreira

**Affiliations:** Coordination of Environmental Dynamics, National Institute of Amazon Research, Av. André Araújo 2936, Petrópolis, Manaus 69067-375, Amazonas, Brazil; Department of Forest Sciences, Soils and Environment, School of Agricultural Sciences, São Paulo State University, Av. 41 Universitária 3780, Altos do Paraíso, Botucatu 18610034, São Paulo, Brazil; Coordination of Environmental Dynamics, National Institute of Amazon Research, Av. André Araújo 2936, Petrópolis, Manaus 69067-375, Amazonas, Brazil; Coordination of Environmental Dynamics, National Institute of Amazon Research, Av. André Araújo 2936, Petrópolis, Manaus 69067-375, Amazonas, Brazil; Department of Forest Sciences, Federal University of Amazonas, Av. Gen. Rodrigo Octávio 6200, Coroado I, Manaus 69080-900, Amazonas, Brazil; Coordination of Environmental Dynamics, National Institute of Amazon Research, Av. André Araújo 2936, Petrópolis, Manaus 69067-375, Amazonas, Brazil; Coordination of Environmental Dynamics, National Institute of Amazon Research, Av. André Araújo 2936, Petrópolis, Manaus 69067-375, Amazonas, Brazil; Department of Forest Sciences, Federal University of Amazonas, Av. Gen. Rodrigo Octávio 6200, Coroado I, Manaus 69080-900, Amazonas, Brazil; Warnell School of Forestry and Natural Resources, University of Georgia, 180 E Green Street, Athens, GA 30602-2152, United States; Warnell School of Forestry and Natural Resources, University of Georgia, 180 E Green Street, Athens, GA 30602-2152, United States; Department of Forest Sciences, Federal University of Amazonas, Av. Gen. Rodrigo Octávio 6200, Coroado I, Manaus 69080-900, Amazonas, Brazil; Form and Function

**Keywords:** drought strategies, saplings, relative growth rate, biomass partitioning, physiological responses, morphological traits, commercial tree species

## Abstract

Extreme events (e.g. severe drought) can hinder the establishment of saplings in tropical forest plantations. To assess the resistance and recovery of three commercially important Amazonian tree species under drought conditions and to identify their key functional strategies for drought response, we conducted a controlled drought experiment exposing saplings of *Bertholletia excelsa*, *Dipteryx odorata*, and *Tachigali vulgaris* to water deficit followed by recovery. *Tachigali vulgaris* (fast-growing species) was more vulnerable to drought, as 80% of the drought-treated plants died. Nevertheless, the individuals who survived demonstrated a rapid recovery of physiological performance following rewatering. *Bertholletia excelsa* and *D. odorata* (slow-growing species) were more resistant to drought stress, as evidenced by lack of mortality in these species. Drought-stressed plants had the lowest growth rates, more biomass allocated to roots and less leaf biomass. The greater biomass allocation to roots in *B. excelsa* and *D. odorata*, together with their more conservative functional traits compared to *T. vulgaris*, appears to play an important role in their lower sensitivity to drought. These species exhibited strategies consistent with drought avoidance. Our results highlight the specific strategies of these species under water-deficit conditions and can help guide decisions on species selection and plantation management for reforestation under climate change scenarios.

## Teaser

A greenhouse experiment was conducted to assess interspecific variation in functional traits of seedlings subjected to drought stress and subsequent recovery. Three commercially important Amazonian tree species—Bertholletia excelsa, Dipteryx odorata, and Tachigali vulgaris—were grown under well-watered and drought-stressed conditions. The fast-growing T. vulgaris exhibited high vulnerability to drought, with 80% mortality under water deficit. In contrast, B. excelsa and D. odorata (slow-growing species) were more resistant to drought stress, as evidenced by the lack of mortality in these species. These species adopted strategies such as conservative functional traits and increased biomass allocation to roots to cope with water limitation. Our findings reveal clear species-dependent patterns in drought responses among Amazonian plants and offer valuable guidance for species selection and plantation management for reforestation and ecological restoration under climate change.

## Introduction

Estimates from the Sixth Intergovernmental Panel on Climate Change (IPCC) indicate that the Amazon region will be severely affected by ongoing climate change (IPCC 2023). These changes include a significant reduction in precipitation, as well as increased temperatures and frequency of intense droughts and floods (Marengo *et al*. 2022). Severe droughts can result in high tree mortality (Rowland *et al*. 2015) and a significant reduction in the productivity of forest plantations (Payn *et al*. 2015). The impacts of drought may be more severe in the initial establishment phase of forest stands since seedlings, with their restricted rooting volume, are more vulnerable to declines in available moisture than are adult trees (McDowell *et al*. 2008).

The magnitude of the impact of drought on forest productivity depends on such factors as the drought length and intensity as well as the strategies adopted by species both to address stress and to resume growth after water deficit. Drought resistance is defined as the ability of plants to respond and acclimate to the harsh environment caused by water-deficit conditions (Fang and Xiong 2015). Drought resistance of plants can be divided into four basic types: drought avoidance, drought tolerance, drought escape (such as transitioning into dormancy in dry season), and drought recovery. The two major strategies are drought avoidance and drought tolerance (Brunner *et al*. 2015, Delzon 2015, Ilyas *et al*. 2020). Drought avoidance is the ability of plants to maintain a favorable water status under mild or moderate drought conditions through morphological and physiological adjustments, such as reducing water loss via rapid stomatal closure and enhancing water uptake by increasing root depth, density, or the root/shoot ratio (Fang and Xiong 2015, Ilyas *et al*. 2020). Drought tolerance refers to the capacity of plants to maintain physiological functions and resist the consequences of dehydration during severe drought through complex regulatory networks involving thousands of genes, as well as metabolic and structural mechanisms such as xylem resistance to embolism and osmotic adjustment (Blum 2005, Fang and Xiong 2015, Ilyas *et al*. 2020).

In addition to detecting stress through different physiological mechanisms, forest species must also be able to adapt to environmental changes and recover their pre-stress conditions once rainfall returns. This behavior is evidenced by drought recovery, which refers to the ability of plants to resume growth after a severe drought (Fang and Xiong 2015, Ilyas *et al*. 2020). Therefore, it is also essential to evaluate the response of the species in the rehydration phase. Although many studies have examined plant responses to drought, the performance of tree species after stress events, especially those native to the Amazon, has been less investigated.

Functional groups such as light-demanding species, fast growing pioneer plants, or N-fixing legume species can respond differently to drought (Mielke *et al*. 2023, Oliveira *et al*. 2021, Yaakobi *et al*. 2023). Although N-fixing legume species generally exhibit higher water use efficiency than other plants (Adams et al. 2016), under water-restricted conditions, non-legume species may adopt drought-avoidance strategies and achieve higher water use efficiency (Yaakobi et al. 2023). There is a trade-off between plant productivity (carbon assimilation and growth) and plant safety (stomatal regulation and water use efficiency) (Yaakobi *et al*. 2023). Slow-growing species often display xylem anatomical traits such as smaller vessel diameters, thicker pit membranes or exhibit more negative turgor loss points, which directly enhance drought resistance and reduce hydraulic failure risk (Li et al. 2015, Oliveira et al. 2021). Therefore, considering species with different ecological strategies is essential when studying plant responses to drought stress.

*Bertholletia excelsa* Bonpl. (Lecythidaceae), *Dipteryx odorata* (Aubl.) Forsyth f. (Fabaceae) and *Tachigali vulgaris* L.G. Silva & H.C. Lima (Fabaceae) are native species of the Amazon with potential for productive and protective forest plantations. *T. vulgaris* is a long-lived pioneer species characterized by rapid growth, nitrogen-fixing capacity, high biomass accumulation, and substantial litter production (Farias *et al.* 2016), making it a strong candidate for charcoal production (Lima *et al*. 2023). *Bertholletia excelsa* and *D. odorata* are non-pioneer (partial shade-tolerant) species with slower growth and multiple uses for timber and nontimber products (such as Brazil nut and coumarin). These species are considered priority for silviculture in the Amazon region (Rolim *et al*. 2019). Although previous studies have examined drought responses in these species (Maltarolo *et al.* 2016, Morais *et al.* 2017, Schimpl *et al.* 2019), evaluating them under the same controlled environmental conditions allows for a more direct comparison of their performance and relative fitness. A better understanding of the strategies for drought recovery will allow the selection of species and plantation designs that are not only highly productive but also most resilient to climate change induced precipitation patterns (Amazonas *et al*. 2018).

We evaluated drought and recovery responses in saplings of three Amazonian tree species to (i) investigate the differences in resistance to and recovery from drought and (ii) identify functional strategies linked to drought resistance mechanisms. We hypothesized that there is a trade-off between drought resistance and recovery and that fast-growing, pioneer species (acquisitive growth strategy) tend to be less resistant, but will recover more quickly compared to slow-growing species (conservative growth strategy). Accordingly, we predicted that fast-growing species such as *T. vulgaris* would exhibit more traits associated to drought recovery than avoidance. Conversely, slower growing species such as *D. odorata* and *B. excelsa* would have traits more related to drought avoidance than recovery.

## Materials and methods

### Plant material and experimental design

The experiment was conducted at the Forest Nursery of the Federal University of Amazonas (3°6′S, 59°58′W), Manaus, Brazil. The experiment was installed in a greenhouse according to a completely randomized design with three species and two treatments (DS = drought stress and WW = well-watered) with 10 replicates (three species × two treatments × ten replicates = sixty plants). We used three evergreen tree species native to the Amazon: the long-lived pioneer species *T. vulgaris* and the partial-shade-tolerant non-pioneer species *Bertholletia excelsa* and *D. odorata* (Swaine and Whitmore 1988, Finegan 1992, Turner 2001, Poorter *et al.* 2006). Among species, we selected plants with similar values of root collar diameter (between 5 and 6 mm) to standardize the plant size. We chose root collar diameter because this attribute is correlated with the total dry mass and the size of the root system (Guimarães *et al.* 2024) and could influence the plants’ response to stress.

In June 2019, 60 saplings (20 per species) cultivated in greenhouse, ∼15 months old, were transferred from 1-l plastic bags to 11-L containers. Fifteen days before the transfer, the substrate (organic black soil regionally known as ‘terra-preta’ or clay) in each container was amended with 32.9 g of dolomitic limestone. To avoid nutrient limitation, we added 2.2 g of N, 2.0 g of K_2_O, 7.7 g of P_2_O_5_ and 2.2 g of FTE-BR12 (1.8% B; 0.8% Cu; 3.0% Fe; 2.0% Mn; 0.1% Mo; 9.0% Zn) when transferring saplings to containers (Gonçalves 1995). The saplings were irrigated once a day for 20 days to acclimatize to the container until the beginning of the experiment. After the acclimation phase, 20 plants of each species were measured, and 10 individuals of similar size were selected to compose each treatment. At the beginning of the experiment, the mean ± standard deviation of height and root collar diameter of plants were 35.71 ± 8.44 cm and 5.90 ± 0.98 mm for *B. excelsa*, 40.17 ± 10.39 cm and 5.87 ± 1.26 mm for *D. odorata*, and 48.13 ± 9.38 cm and 5.11 ± 0.85 mm for *T. vulgaris*, respectively.

Irrigation was performed once a day to maintain moisture levels at field capacity, i.e. when water percolated through the container to the counter and soil was saturated. The values of soil moisture during the experiment phases are shown in Supplementary Table S1. For each species, 10 plants were kept under constant irrigation (well-watered), while irrigation was suspended for another group of 10 plants (drought-stressed) until maximum stress was reached—defined as the point at which most drought-treated plants exhibited photosynthesis rates close to zero (35 days after water suspension). For this reason, gas exchange monitoring was performed at short intervals. After the stress phase, irrigation was resumed until most of the plants obtained photosynthesis values similar to those of plants in the control group. The plants were placed on benches 1.30 m above the ground. Irrigation treatments were separated by 2 m and positioned at the ends of each bench. Within each treatment, species were arranged sequentially along with their replicates. To minimize positional effects, the arrangement of species within each treatment was alternated every 5–7 days. However, pots from the drought treatment were never placed in areas receiving manual irrigation or occasional wetting to maintain treatment consistency.

### Survival, relative growth rates, accumulation, and partition of biomass

The survival, growth, and accumulation and partition of biomass were obtained at the end of the experiment. The percentage of survival of each species in the treatments was obtained by the number of live plants divided by the initial number of plants × 100. The total height and root collar diameter were measured for all the plants at the beginning (July 2019) and end (October 2019) of the experiment. The relative growth rates were calculated as follows: RGR_X_ = (lnX_2_ − lnX_1_)/(T_2_ − T_1_), according to Hunt (1990), where X_1_ = initial height or root collar diameter, X_2_ = height or root collar diameter at the end of the experiment and T_2_ − T_1_ = interval between measurements (62 days). We measured leaf number (LN), total leaf area (TLA), leaf dry mass (LDM), stem dry mass (SDM), aboveground biomass (AB = LDM + SDM), root dry mass (RDM), and total dry mass (TDM = LDM + SDM + RDM). Leaves, stems, and roots were separated, and the roots were washed to remove soil particles. The leaf size and total leaf area of each plant were measured using a leaf area meter (CI-202, CID, Inc. Camas, WA, USA). For the compound leaves of *D. odorata* and *T. vulgaris*, the leaflets were counted as leaves. All the fresh matter (leaves, stems, and roots) was dried at 65°C for 72 hours, and the dry mass was recorded using a digital scale with a precision of 0.01 g. For compound leaves, the rachis was included in the dry leaf mass. The seeds of *B. excelsa* are a source of reserves for the plant and the plant can remain with the seed for months or years after germination, depending on the growth rate. As a result, the seed was not considered part of the dry mass. The following biomass partitions were calculated: leaf mass fraction (LMF; LDM/TDM), leaf area ratio (LAR; TLA/TDM), stem mass fraction (SMF; SDM/TDM), root mass fraction (RMF; RDM/TDM), and the root/shoot ratio (R/S; RDM/AB).

### Functional leaf traits

All measurements were performed on fully expanded leaves with no signs of pathogen infection or herbivory. All measurements were made on one leaf or leaflet per plant, except for chlorophyll *a* fluorescence, which was measured on two leaves or leaflets per plant. All plants were evaluated, except those that did not have leaves at the time of measurement. The non-destructive measurements (gas exchange and chlorophyll *a* fluorescence) were taken at 7-day intervals during the drought phase (six measurement dates) and at 3- to 7-day intervals during the rewatering phase. To allow repeated measurements, the leaves used for non-destructive evaluations were marked with colored tape and followed throughout the experiment. If a marked leaf was damaged or abscised, it was replaced with a similar leaf. For destructive measurements, different leaves were collected from the same plants under comparable conditions to those of the marked non-destructive leaves

The environmental conditions were monitored for 20 days throughout the experiment from 0700 to 1200 h to characterize the period of data collection. The mean ± standard deviation values for greenhouse conditions during physiological measurements were PPFD = 412.7 ± 231.7 µmol m^−2^ s^−1^, air temperature = 30 ± 4°C, air humidity = 67.9 ± 9.2%, and vapor pressure deficit = 1.47 ± 0.72 kPa (for details, see Supplementary Table S2).

Stomatal traits were analyzed once, at the beginning of the experiment. For stomatal density and size (length of the guard cell), two samples (one on the right and one on the left of the central region of the leaf) were obtained from each leaf. These samples were subjected to dissociation of the epidermis with hydrogen peroxide and acetic acid (Franklin 1945). The samples were stained with safranin and dehydrated in an alcohol series, after which the slides were fixed in glycerin. Using a digital camera attached to a light microscope (Carl Zeiss Microscopy GmbH, Germany), images were taken in two fields for each sample (four fields per leaf), and on average, thirty stomata per leaf were measured. Only the abaxial side was used since all species have hypostomatic leaves. The images were analyzed using ImageJ software version 2.0.0-rc-44/1.50e (Abràmoff et al. 2004). The evaluation of stomatal traits after stress was planned to be performed on mature leaves shed during the rehydration phase to observe possible adaptive changes. However, none of the leaves reached tissue maturity by the end of the experiment, so only the analysis for species characterization was maintained.

LWP was measured between 6:00 and 7:00 a.m. (sunrise occurs at ∼ 0600 h a.m.) with a pressure chamber (model 3115; Soil Moisture Equipment Corp., USA) (Scholander *et al*. 1965). Measurements were made in three occasions: beginning, maximum stress and at the final of the experiment (after rewatering). When the pressure value without exudation approached the maximum supported by the instrument (−4 MPa), this LWP value was recorded.

Each set of leaf gas exchange measurements was taken on two consecutive days between 7:30 a.m. and 10:30 a.m. using a portable open system infrared gas analyzer (LI-6400XT, LI-COR, Lincoln, USA), and the net photosynthetic rate (*P*_N_), dark respiration (*R*_d_), and stomatal conductance (*g*_s_) were obtained. The fixed parameters were flow = 400 µmol s^−1^, CO_2_ = 400 µmol mol^−1^, leaf temperature = 31°C, vapor pressure deficit = 1.9 ± 1 kPa, and photosynthetic photon flux density (PPFD) = 2000 µmol m^−2^ s^−1^. *R*_d_ was measured at a PPFD equal to 0 µmol m^−2^ s^−1^. Intrinsic water use efficiency (*WUE*_i_) was calculated as the ratio of *P_N_* to *g*_s_ (Nogueira *et al*. 2004). The order plants were measured was alternated (mixing species and treatments) to avoid a sequential bias in the measures but considering the critical stomatal closing times for each species. As *D. odorata* and *B. excelsa* closed their stomata earlier, the measurement of their individuals was concentrated more early in the morning, but never all in sequence. With stress progression, the measurements started with the plants in the drought stress treatment group.

The chlorophyll *a* fluorescence was measured on two leaves per plant (one leaf was the same as gas-exchange measurements) between 0800 and 0900 h hours using a portable fluorimeter (PEA, MK2–9600—Hansatech, Norfolk, UK). Using leaf clips, a 4-mm diameter area of each leaf was dark-adapted for 30 minutes to allow complete oxidation of the photosynthetic electron transport chain. Then, the leaves were exposed to a pulse of saturating light intensity of 3000 µmol m^−2 ^s^−1^ (650 nm) for 1 s. The JIP test was used to determine the maximum quantum yield of primary PSII photochemistry (*F*_v_/*F*_m_) and the total performance index (*PI*_total_) (Strasser *et al*. 1995, 2010).

### Data analyses

Only for stomatal traits, species were considered a factor and were compared using ANOVA and Tukey’s *post hoc* test (*α* = 0.05). For each species, we used unpaired *t* tests or Mann–Whitney *U* tests (*α* = 0.05) to compare the two treatments (well-watered and drought stressed) at the end of experiment (biomass) or during the three phases of the experiment (LWP). Generalized linear mixed models (GLMMs) were applied to evaluate the effect of time (12 days of measurements) on photosynthetic traits as dependent on the treatments. We added the individual as a random factor due temporal non-independence (autocorrelation) of the measures. In GLMM, random factors help improve the model’s ability to make inferences and predictions, while accounting for this nonindependence. GLMM analyses were conducted using the nlme package version 3.1-168 and the following model structure: model < − lme (response ∼ factor(time) * treatment, data = cast, random = ∼ 1 | ind, correlation = corAR1(form = ∼ time | ind)). This function specifies an autoregressive correlation structure and is used in conjunction the individual random effect to account for the temporal correlation structure in the data. We compare treatments at each time or within each treatment over time (when there was a difference between treatments at the initial time or change in the behavior of the control treatment) by Tukey *post hoc* test using the emmeans package version 2.0.0. In cases where few individuals remained for analysis (either due to mortality or lack of leaves), no statistical comparison was made. Analyses were performed in R software version 4.4.1 (R Core Team 2022).

## Results

### Survival, growth, accumulation, and partitioning of biomass

**Figure 1:**
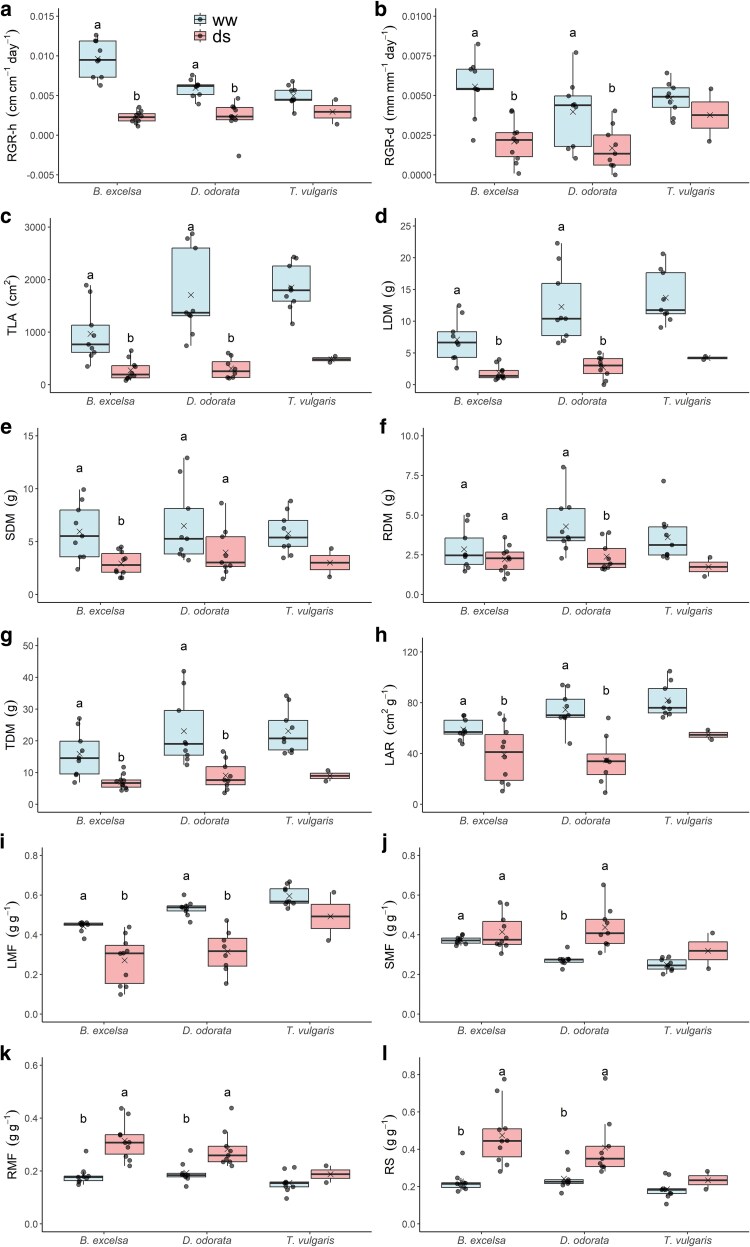
Boxplot showing the relative growth rates, total accumulated biomass, and biomass partitioning of three Amazonian forest species after 62 days of the experiment. ww = well-watered and ds = drought-stressed plants. Relative growth rates according to height (RGR-h) and diameter (RGR-d), total leaf area (TLA), stem dry mass (SDM), root dry mass (RDM), total dry mass (TDM), leaf dry mass (LDM), leaf mass fraction (LMF), leaf area ratio (LAR), stem mass fraction (SMF), root mass fraction (RMF), and the root/shoot ratio (R/S) were calculated. Different letters indicate significant differences between treatments for each species (*P* < .05). In the boxes, lines indicate the medians and × indicates the means. For *Tachigali vulgaris* under drought stress, the values correspond only to living saplings (*n* = 2).

*Tachigali vulgaris* was the only species that experienced mortality (80%). *Bertholletia excelsa* showed the greatest reductions in height growth (76% versus 61% for *D. odorata* and 41% for *T. vulgaris*). The only two surviving plants of *T. vulgaris* showed the lowest reductions in diameter growth (22%) compared to 62% for *B. excelsa* and 58% for *D. odorata*. We emphasize that although only two individuals of *T. vulgaris* survived, we consider it important to include comparisons for this species. However, we recommend that these should be taken with caution. Drought-stressed plants accumulated less biomass than the well-watered plants (Fig. 1d–g), especially in leaves (Fig. 1c and d). There was a significant decrease in number of leaves of drought-stressed plants (Supplementary Figure S1), DS plants had 69% less leaves than WW plants. On average, the reduction in stem dry mass in *B. excelsa* was 46% (Fig. 1e). However, *D. odorata* had no statistically significant differences in stem dry mass, and *B. excelsa* had no difference in root dry mass (Fig. 1f). The species exhibited differences in biomass partitioning under drought stress (Fig. 1h and l). *Dipteryx odorata* had the greatest reductions in leaf area ratio (−59%) and leaf mass fraction (−47%) and the greatest increase in stem mass fraction (+60%). *Bertholletia excelsa* did not significantly differ in stem mass fraction between the treatments (Fig. 1j). *Bertholletia excelsa* had the highest increase in the root mass fraction (+71%), while *D. odorata* and *T. vulgaris* had increases of 46% and 21%, respectively. *Bertholletia excelsa* and *T. vulgaris* showed the highest and lowest increases in the R/S ratio, 109% and 26%, respectively. An increase of 69% in the R/S ratio was detected for *D. odorata*.

### Leaf traits

*Dipteryx odorata* and *T. vulgaris* had stomatal density values lower than those of *B. excelsa* (Table 1). *B. excelsa* also presented the lowest stomatal size (length of the guard cell). *T. vulgaris* also exhibited trichomes on the abaxial side (Supplementary Figure S2).

**Table 1:** Mean values ± standard errors of stomatal traits of three Amazonian forest species before treatments.

Trait	*Bertholletia excelsa*	*Dipteryx odorata*	*Tachigali vulgaris*	*F (P)*
Stomatal density (mm^−2^)	407 ± 19 a	260 ± 10 b	230 ± 8 b	52.41***
Stomatal size (µm)	17.47 ± 0.26 c	24.53 ± 0.23 a	21.53 ± 0.17 b	249.39***

Different letters indicate significant differences among species according to Tukey’s *post hoc* test (*P* < .05).

^***^ *P* < .001.

**Figure 2:**
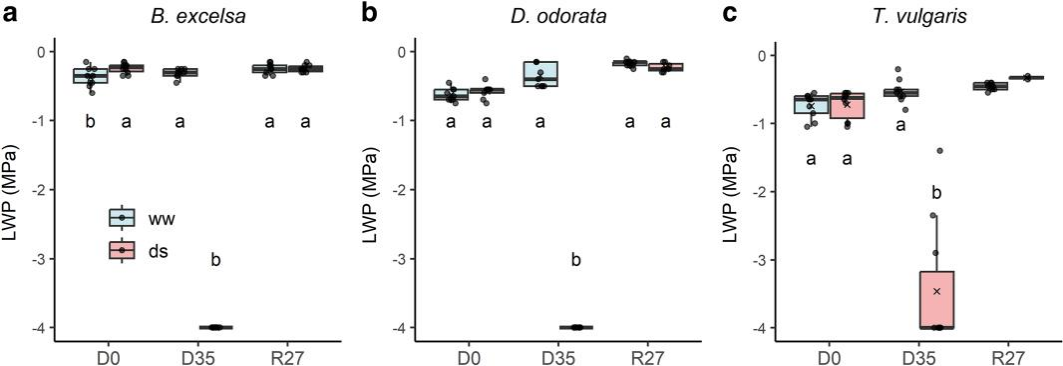
Mean values ± standard errors of changes in leaf water potential (LWP) of three Amazonian forest species in three times: initial (D0), 35 days of drought (D35) and after 27 days of rewatering (R27). ww = well-watered and ds = drought-stressed plants. For the DS treatment, D35 corresponded to the maximum stress phase, and for the WW treatment, R27 corresponded to day 62. Different letters indicate significant differences between treatments for each period according to unpaired tests (*P*  *<* .05). In the box, lines indicate the medians, and × indicates the means. For *Tachigali vulgaris* under drought stress, the values at R27 correspond to only surviving saplings (*n* = 2).

LWP in drought-treated plants reached −4.0 MPa in *B. excelsa* and *D. odorata*, and −3.47 MPa in *T. vulgaris* (Fig. 2). We note that −4.0 MPa was the lower detection limit of the equipment, so actual values may have been lower. After rewatering, all species showed recovery, and by 27 days post-rehydration, drought-treated plants reached LWP values similar to the control (−0.25 MPa for *B. excelsa*, −0.23 MPa for *D. odorata*, and −0.33 MPa for *T. vulgaris*).

### Gas exchange and water use efficiency

**Figure 3:**
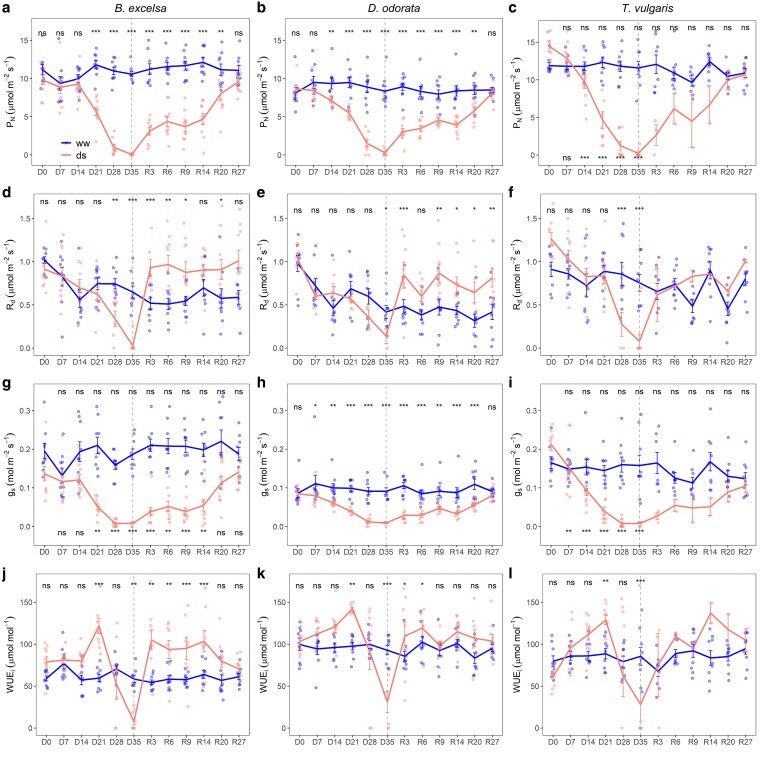
Mean values ± standard errors of gas exchange for three Amazonian forest species during the time of treatment initiation: 35 days of drought (D0–D35) and 27 days of rewatering (D35 or R0–R27). ww = well-watered and ds = drought-stressed plants. For the well-watered treatment, R3, R6, R9, R14, R20, and R27 corresponded to days 38, 41, 44, 49, 55, and 62, respectively. The dashed line represents the change in the water regime. Net photosynthesis (*P*_N_), dark respiration (*R*_d_), and stomatal conductance (*g*_s_) and intrinsic water use efficiency (*WUE*_i_). Differences for treatments on each day or over time within each treatment according to *post hoc* Tukey are inserted as *P < .05; **P < .01; ***P < .001; n.s. (not significant) *P* ≥ .05. When comparisons are made over time for the same treatment, the significance of the WW treatment is on the top and that of the DS treatment on the bottom. For *T. vulgaris* in the rewatering stage, the values correspond to only surviving saplings (*n* = 2).

Overall, the values of photosynthesis declined to near zero at 35 days after the suspension of irrigation, which is the point which we consider maximum stress. The physiological traits of gas exchange were affected in different times (Fig. 3). Changes occurred earlier for *g*_s_ (7–21 days), *P*_N_ (14–21 days), *WUE*_i_ (21 days), and *R*_d_ (28–35 days). Among species, *T. vulgaris* showed faster changes and *B. excelsa* showed later changes. For *g*_s_ of *D. odorata, P*_N_ and *g*_s_ of *T. vulgaris* comparisons were made over time relative to the initial time due statistically significant differences between treatments in day zero.

For *B. excelsa*, significant difference between treatment and control group only occurred when physiological variables (*P*_N_, *R*_d_ and *g*_s_) reduced around 52%–62%. For *D. odorata,* reductions in *g*_s_ (28%) and *P*_N_ (24%) occurred early (7 and 14 days, respectively) but changes in *R*_d_ were late, only at maximum stress, with a 66% of the reduction in comparison to the control group. For *T. vulgaris*, changes in *P*_N_ and *g*_s_ occurred at the same time for *D. odorata*, but reductions were higher for *T. vulgaris*—37% for *P*_N_ and 43% for *g*_s_. *R*_d_ of DS plants of *T. vulgaris* reduced 71% on the 28th day, at the same time for *B. excelsa*. For all species, *WUE*_i_ differed between treatments after 21 days, but with an increase in the values for DS plants, especially for *B. excelsa* (105%) in comparison with the other two (45%). Although *D. odorata* had the lowest initial values of *P*_N_ and *g*_s_, this species only reached values of *P*_N_ close to zero at maximum stress.

Species recovered values of *P*_N_ after 27 days of rewatering (Fig. 3a and b). However, the response velocity differed between species; after 9 days of rewatering, while *B. excelsa* recovered 32% of the *P*_N_, *D. odorata* recovered 57%. The only two surviving *T. vulgaris* plants recovered 98% of *P*_N_ after 20 days of rewatering (Fig. 3c). For *B. excelsa* and *D. odorata,* values of *R*_d_ in DS plants increased in the rewatering phase (Fig. 3d and e). *Bertholletia excelsa* recovered values of *g*_s_ 7 days before *D. odorata*, despite *D. odorata* having the lowest *g*_s_ values among the species. DS plants increase *WUE*_i_ in the rewatering phase, but the higher values of *WUE*_i_ for DS than WW plants remained until 9 days for *D. odorata* and 20 days for *B. excelsa*.

### Chlorophyll *a* fluorescence

**Figure 4:**
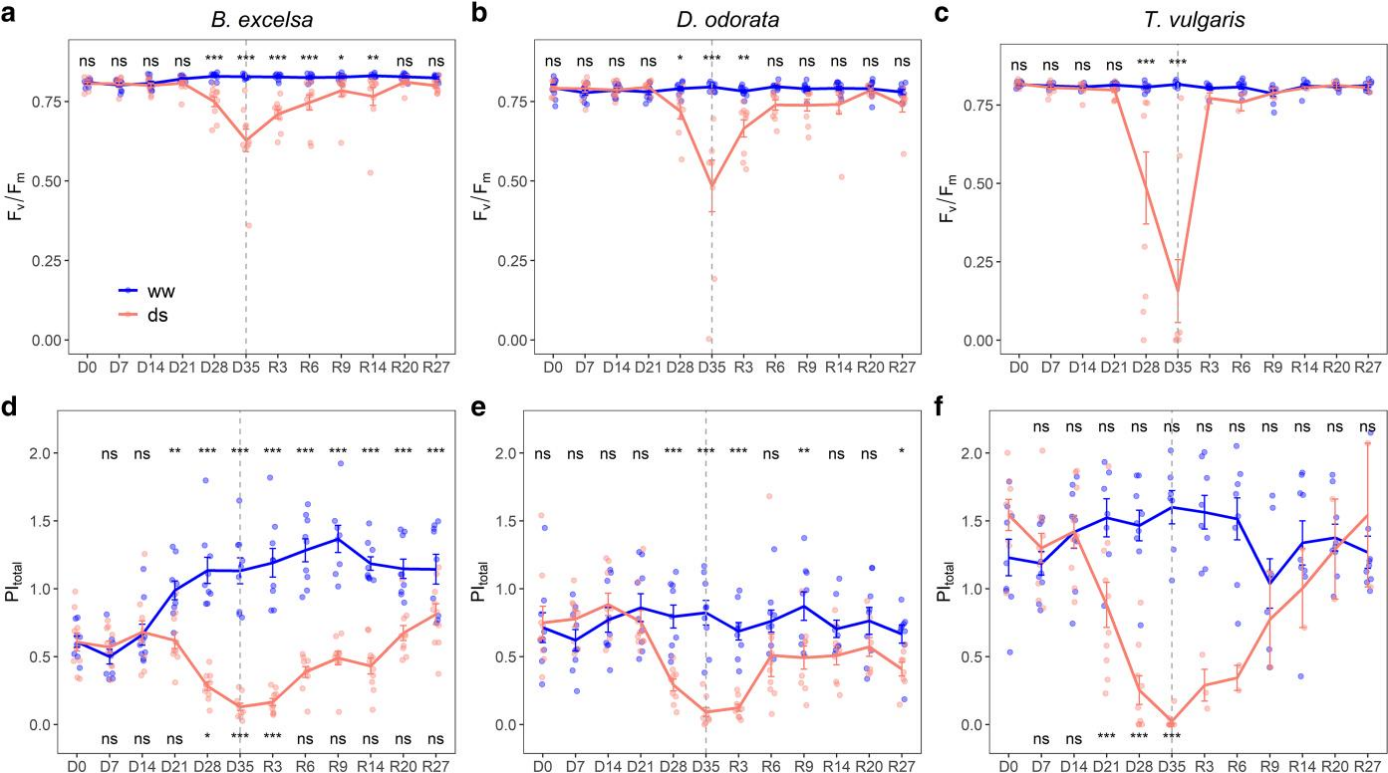
Mean values ± standard errors of the maximum quantum yield of PSII (*F*_v_/*F*_m_) and total performance index (*PI*_total_) of three amazonian forest species during the time of treatment initiation: 35 days of drought (D0–D35) and 27 days of rewatering (D35 or R0–R27). ww = well-watered and ds = drought-stressed plants. For the well-watered treatment, R3, R6, R9, R14, R20, and R27 corresponded to days 38, 41, 44, 49, 55, and 62, respectively. The dashed line represents the change in the water regime. Differences for treatments on each day or over time within each treatment according to *post hoc* Tukey are inserted as *P < .05; **P < .01; ***P < .001; n.s. (not significant) *P* ≥ .05. When comparisons are made over time for the same treatment, the significance of the WW treatment is on the top and that of the DS treatment on the bottom. For *T. vulgaris* in the recovery stage, the values correspond to only surviving saplings (*n* = 2).

For all the species, differences in *F*_v_/*F*_m_ occurred only on the 28th day, while in *PI*_total_ they occurred between 21 (*T. vulgaris*) and 28 days (*B. excelsa* and *D. odorata*) (Fig. 4). At maximum stress, *F*_v_/*F*_m_ in DS plants reduced (0.628 for *B. excelsa*, 0.484 for *D. odorata* and 0.156 for *T. vulgaris*) compared to control (0.828 for *B. excelsa*, 0.796 for *D. odorata* and 0.816 for *T. vulgaris*) (Fig. 4a–c). The low values of *F*_v_/*F*_m_ for *T. vulgaris* are associated to the oxidative stress of the leaves that occurred between 21 and 28 days (Supplementary Figure S3). Because *PI*_total_ of the reference group (well-watered plants) of *B. excelsa* and *T. vulgaris* varied during stress progression, we compared the measurements in the time for each treatment with the initial values at day 0. At maximum stress, *PI*_total_ in DS plants reduced (0.130 for *B. excelsa* and 0.024 for *T. vulgaris*) compared to initial values (0.608 for *B. excelsa* and 1.744 for *T. vulgaris*) (Fig. 4d and e). For *D. odorata*, *PI*_total_ was lower in DS plants (0.091) compared to WW plants (0.823) (Fig. 4f).

*Bertholletia excelsa* recovered values of *PI*_total_ after 6 days of rewatering but recovered *F*_v_/*F*_m_ only after 20 days. *D. odorata* recovered *F*v*/F*m more quickly than *B. excelsa* (within 6 days), but *PI*total did not fully recover even after 27 days of rewatering (Fig. 4e). For *T. vulgaris*, the intense oxidation of the leaves and death of the plants compromised the analyses, but the two living plants showed values near the control for *F*_v_/*F*_m_ (after 3 and 6 days) and near the beginning of the experiment for *PI*_total_ (after 20 days) (Fig. 4f).

## Discussion

In our study, we assess the resistance and recovery of three commercially important Amazonian tree species under drought conditions and identify functional strategies linked to drought resistance mechanisms. As we predicted, *T. vulgaris* was more vulnerable to drought than the other species, as 80% of the drought treated plants died, whereas the few plants that survived exhibited fast recovery of physiological parameters after rewatering. *Bertholletia excelsa* and *D. odorata* were more resistant to drought stress, as evidenced by the lack of mortality in these species. Our results, which show a greater allocation of biomass to roots in drought-treated plants of *B. excelsa* and *D. odorata* also because of the reduction in leaf biomass, reinforce and expand upon existing evidence in the literature that identifies increased root investment as an important drought resistance strategy in forest species. In the following sections, we delve into the primary findings.

### Interspecific variation in morphological and physiological responses to drought stress

The high mortality of *T. vulgaris* seedlings after the drought experiment clearly indicates this species is much more vulnerable than *D. odorata* and *B. excelsa*. Drought-stressed plants of *T. vulgaris* exhibited the lowest reduction in growth, the highest total leaf area, the lowest root mass fraction, and the lowest increase in the root-to-shoot ratio during drought conditions (Fig. 1 and Supplementary Table S3). This strategy resulted in higher transpiration surface and lower investment in water absorption. *T. vulgaris* has a shallow root system and can be found in dry regions such as the Brazilian savanna (Cerrado) (Jackson *et al*. 1999). Recent evidence for high mortality of *T. vulgaris* trees has been shown for southern Amazonian edge forests subjected to intense dry seasons (Reis *et al.* 2022). This species allocates a small fraction of carbon to root growth (Guimarães *et al*. 2025), which can help to explain the high mortality of the plants during drought stress. However, the evidence of leaf oxidation in DS plants of *T. vulgaris* (Supplementary Figure S3) suggests severe photo-oxidative damage leading to leaf senescence following 20 days of water suspension, contributing to high mortality.

The preferential carbon allocation to roots under water deficit in *B. excelsa* resulted in lower investment in aboveground biomass, reducing height growth in DS plants. Despite the reductions in leaf biomass for all the species, the strategies used by *B. excelsa* to increase the root biomass and improve water uptake may have reduced the risk of death. Greater root-to-shoot ratios likely enhance a plant's ability to access soil water later in the growing season, reducing the risk of water deficit by increasing the supply of water to aboveground portions of the plant (Valliere *et al*. 2019). This preferential investment in roots is part of the strategy to avoid drought (Ilyas *et al*. 2020). Changing allocation too quickly might then result in suboptimal growth after the water supply is restored (Poorter *et al*. 2012), impacting species resilience. *Dipteryx odorata* had intermediate reductions in growth and the greatest reductions in leaf area ratio and leaf mass fraction, probably due to the reductions in leaf number as part of the drought avoidance strategy (Supplementary Figure S1).

*Bertholletia excelsa* plants showed late visual signs of leaf dehydration (∼30 days under drought), although they reached very negative leaf water potentials (below −4 MPa). This delay in dehydration for *B. excelsa* must have contributed to the delay in negative effects in gas exchange (Fig. 3). The ability of plants to resist dehydration is associated with drought tolerance and leaf carbon investment (Zhu *et al*. 2018). Although we have not measured the turgor loss point, *B. excelsa* has previously demonstrated the ability to strongly adjust osmotic content. A study of *B. excelsa* plants under 58 days of drought stress revealed soluble sugar and starch levels indicative of osmotic function supporting decreased leaf water potential (Schimpl *et al*. 2019).

Overall, the water use efficiency of the DS plants increased (Fig. 3j–l). An increase in water use efficiency indicates that stomatal conductance is reduced more than photosynthesis, mainly during the stress phase, as a plant strategy to improve photosynthetic performance by decreasing the use of limited resources (Schimpl *et al*. 2019, Leite *et al*. 2022). Despite the similar stomatal density between *T. vulgaris* and *D. odorata* (Table 1), the stomatal size varied, which may account for the different drought responses of the two species. Smaller stomata have a greater surface area to volume ratio, facilitating faster movements in response to changing leaf hydration (Henry *et al*. 2019). *T. vulgaris* drastically reduced *P*_N_ and *g*_s_, while *D. odorata* controlled this decline throughout the stress period. The slow decline in *g*_s_ of *D. odorata* must have favored the maintenance of growth (lower reductions in RGRs than for *B. excelsa*). In terms of stomatal safety and efficiency, fast-strategy species tend to reduce stomatal conductance more rapidly under water deficit, whereas slow-strategy species can maintain leaf functioning under higher levels of water stress (Guillemot *et al*. 2022).

The reductions in *P*_N_ were related to nonstomatal limitations only in the last 7 days of the drought (Figs. 3, 4). *Bertholletia excelsa* maintained the best photochemical performance during drought. An early decrease of the PI_total_ in *T. vulgaris,* and the lowest values of *F*_v_/*F*_m_ can be due to the photo-oxidative damage leading to leaf senescence observed only in this species. A reduction in *F*_v_/*F*_m_ occurs when leaf dehydration reaches a more critical level (Mielke *et al*. 2023). In addition, PI_total_ has been shown to be more sensitive to drought stress than *F_v_/F_m_* (Stirbet *et al*. 2018). For *B. excelsa* and *D. odorata*, these reductions in photochemical performance likely resulted in photoinhibition (a net decrease in the efficiency of photosynthesis due to excess absorbed energy that plants are not using for carbon assimilation), rather than photoinactivation (the structural degradation or loss of PSII reaction center functionality) (Zuo 2025). Another explanation is that since photosynthesis is limited by drought (stomatal limitations), plants might accumulate reducing power when exposed to light to avoid possible damage to photosystems (Leite *et al*. 2022).

### Interspecific variation in morphological and physiological responses during recovery

A faster recovery may enable a competitive advantage by resuming growth after drought (Frosi *et al*. 2017). For *B. excelsa*, the increase in water use efficiency in DS plants even after rewatering indicates plasticity and can be considered an individual strategy to further increase plant tolerance to stress; this change may be associated with its water-saving strategy (Forner *et al*. 2018). The production of new leaves and increased WUE provide short-term advantages to *B. excelsa* after a drought event, contributing to growth resumption during rewatering or during the next wet season under field conditions. The stomatal leaf traits of *B. excelsa* (those with high density of small stomata) should provide better stomatal control, which could improve the species’ responsiveness during rewatering.

The DS plants of *D. odorata* and *B. excelsa* had higher respiration rates than WW plants (Fig. 3). This increase in respiration decreases carbon use efficiency at the leaf level and can reduce growth after rains return. *Dipteryx odorata* had a faster recovery of *P*_N_. *Dipteryx odorata* and surviving plants of *T. vulgaris* recovered *F*_v_/*F*_m_ more quickly than *B. excelsa*. *F*_v_/*F*_m_ can also indicate differences in the recovery capacity of species after a period of moderate or severe drought stress (Frosi *et al*. 2017) and has been proposed to reflect drought resilience through photochemical functions (Fortunel *et al*. 2023). The recovery of photochemical performance of *B. excelsa* and *D. odorata* may be another indication of the defense mechanism (accumulation of reducing power), which protects the photosystem from photoinhibition and allows recovery after irrigation is resumed (Leite *et al*. 2022). The full recovery of leaf water potential and gas exchange rates in *B. excelsa* and *D. odorata* after 27 days indicates that these species were able to restore the physiological conditions necessary to support continued growth. An increase in photosynthesis when rains return is indicative of resilience to one-time extreme climatic events in Amazonian forests (Santos *et al*. 2018). In addition to the increase of the root/shoot ratio, which has been shown globally to relate to drought tolerance (Puglielli *et al.* 2021), rapid rehydration suggested greater connectivity of the xylem network. This connectivity suggests that a reduction in stem hydraulic conductance may not have occurred at a level that restricted water transport, allowing the possibility for normal physiological function to resume (Rehschuh *et al*. 2020).

Although two individuals of *T. vulgaris* survived the drought and were able to partially recover their physiological performance—indicating some intraspecific variability in drought response—the high overall mortality observed suggests that *T. vulgaris* cannot be considered drought-resistant.

### Potential strategies for drought resistance and recovery

*Bertholletia excelsa* and *T. vulgaris* are more acquisitive, that is, they have characteristics that favor growth, such as higher stomatal conductance, photosynthesis and photochemical performance. *Bertholletia excelsa* displays a combination of both acquisitive and conservative traits, challenging the traditional trade-off between rapid growth and drought tolerance. These results further support its characterization as an exceptional tree species that combines relatively fast growth with low mortality (Sugiyama et al. 2024, Modolo et al. 2025). *Dipteryx odorata* was more conservative, that is, it has characteristics that favor survival, such as lower values for gas exchange traits. Drought-stressed plants had greater biomass allocated to roots, which was associated with a reduction in leaf biomass. A higher RMF and R/S ratio in DS plants of *B. excelsa* and *D. odorata* are mechanisms of drought avoidance. Changing allocation to roots (e.g. RMF and the R/S ratio) favors the survival of plants during drought (Poorter and Markesteijn 2008) and can result in suboptimal growth after the water supply is restored (Poorter *et al*. 2012). The drought avoidance mechanisms demonstrated by *D. odorata* included reducing water loss and reducing leaf area (the greatest reductions in LAR and LMF) (Fig. 1).

A fast-slow continuum can explain the interspecific differences in response to drought (Eller *et al*. 2018, Oliveira *et al*. 2021). *Dipteryx odorata* has more conservative strategies, such as lower photosynthesis, transpiration and stomatal conductance and greater water use efficiency (Fig. 3) and may be considered a slow species. *T. vulgaris*, in contrast, is an acquisitive species with fast growth and has high rates of photosynthesis and stomatal conductance (Guimarães *et al*. 2018), which may demand additional resources, such as water, making it more susceptible to hydraulic failure. Fast-growing trees have a greater risk of hydraulic failure (lower xylem safety margins) than slow-growing trees (Eller *et al*. 2018). A recent study showed that in *T. vulgaris*, the combination of persistent transpiration during drought and high hydraulic conductivity can increase the risk of xylem cavitation and hydraulic failure (Salomon *et al.* 2025). Although the presence of non-glandular trichomes (Supplementary Figure S3) is related to water conservation, as reported by Ribeiro-Júnior *et al*. (2023), *T. vulgaris* presented more functional characteristics associated with vulnerability to drought.

### Implications for silviculture adaptive procedures in the Amazon

Extreme climatic events (e.g. severe drought) have been found to affect forest plantations globally (Payn *et al*. 2015). To enhance reforestation success in areas prone to droughts (some of which are due to climate change scenarios), it is crucial to consider species-specific traits and habitat requirements before planting, with a focus on selecting drought-tolerant species (Mugwedi *et al*. 2021). Knowledge of the strategies adopted by species to address and overcome drought stress is important for decision-making regarding the management of these species in a scenario of greater frequency and intensity of drought in the Amazon.

We know that nursery responses are not always correlated with field responses, especially in the Amazon, where most areas experience no more than 15 days without rain, and planting is recommended during the rainy season. Under field conditions, plants may require a longer dry period or a combination with another stress factor to achieve the same mortality rates as this study. Therefore, these results are important but limited.

For *T. vulgaris,* low productivity under drought may restrict the use of this species in monocultures for energy purposes in regions with water restriction (e.g. a dry season of 5–8 months) (Tonini *et al*. 2018). However, conservative species can act as facilitators in mixed plantations, creating favorable environmental conditions for the establishment of less resistant and more water-demanding species, such as *T. vulgaris*. Niche complementarity effects (i.e. functional diversity) among plant species are becoming increasingly important for annual forest growth under increased water limitation (Lammerant *et al*. 2023). Another possibility is to establish the species in enrichment plantations (more favorable microclimatic conditions) or in monocultures with lower density. Therefore, we cannot currently recommend it for high-density monoculture planting due to apparent susceptibility of the species to extreme drought. For the most drought-resistant species, *D. odorata* and *B. excelsa*, the capacity to resist drought due to avoidance and tolerance mechanisms reinforces the selection of these two species as priorities for planting in the Amazon.

## Conclusion

This study provides evidence on the functional responses of Amazonian tree species to drought stress and subsequent recovery. *Tachigali vulgaris* (fast-growing species) was more vulnerable to drought, whereas the few plants that survived exhibited fast recovery of physiological parameters after rewatering. **Bertholletia* excelsa* and *D. odorata* (slow-growing species) were more resistant to drought stress, as evidenced of lack of mortality in these species. The higher allocation of biomass to roots in *B. excelsa* and *D. odorata* associated with more conservative traits than *T. vulgaris* appears to play a crucial role in the sensitivity of these Amazonian commercial species to drought. *Bertholletia excelsa* and *D. odorata* exhibited strategies related to drought avoidance. The total recovery of *B. excelsa* and *D. odorata* highlights their resilience and suitability for large-scale planting.

## Supplementary Material

plaf073_Supplementary_Data

## Data Availability

Raw data and R code are available online at https://data.mendeley.com/preview/f492hsc82d?a=819c59d0-e8dc-4981-87ec-3aeb41ad0a49.

## Abbreviations

AB: aboveground biomass
DS: drought stress
*E*: transpiration rate
*F*_v_/*F*_m_: maximum quantum yield of primary PSII photochemistry
*g*_s_: stomatal conductance
H: height
LAR: leaf area ratio
LDM: leaf dry mass
LMF: leaf mass fraction
LN: leaf number
LWP: leaf water potential
*PI*_total_: total performance index
*P*_N_: net photosynthetic rate
PPFD: photosynthetic photon flux density
RCD: root collar diameter
*R*_d_: dark respiration
RDM: root dry mass
RGR-d: relative growth rate in root collar diameter
RGR-h: relative growth rate in height
RMF: root mass fraction
R/S: root/shoot ratio
SDM: stem dry mass
SM: soil moisture
SMF: stem mass fraction
TDM: total dry mass
TLA: total leaf area
*WUE*_i_: intrinsic water use efficiency
WW: well-watered
