# Supplementary material for: Interspecific variation of functional traits in saplings of three Amazonian species under drought stress and recovery
Source: AoB Plants. 2026 Jan 8;18(1):plaf073. doi: 10.1093/aobpla/plaf073 (PMC12833982; doi:10.1093/aobpla/plaf073)
Supplement: plaf073_Supplementary_Data [file plaf073_supplementary_data.zip › Supplementary material.docx]

**Supplementary material**

Table S1. Mean values ± standard errors of changes in soil moisture (w/w in %) over 62 days of the experiment (N = 3). WW = well-watered, DS = drought stress. 0 = initial conditions, 35 = maximum drought stress and 62 = after rewatering. Different letters in the line indicate significant differences between treatments for each period according to unpaired t tests (P < 0.05).

| Day | WW | DS |
| --- | --- | --- |
| 0 | 41.2 ± 0.7 a | 44.1 ± 4.8 a |
| 35 | 34.4 ± 1.3 a | 5.9 ± 1.2 b |
| 62 | 43.6 ± 1.2 a | 33.5 ± 1.7 b |

Table S2. Mean ± standard deviation of the photosynthetic photon flux density (PPFD), air temperature and relative humidity and vapor pressure deficit (VPD) measured in 20 days during the experimental period (July to October 2019). For the PPFD, the values that characterize each time point correspond to the 30 minutes before and after each hour.

| Hour | PPFD (µmol m^−2^ s^-1^) | Air temperature (°C) | Relative humidity (%) | VPD (kPa) |
| --- | --- | --- | --- | --- |
| 07:00 | 78.52 ± 31.97 | 24.93 ± 0.58 | 77.50 ± 2.50 | 0.71 ± 0.07 |
| 08:00 | 187.37 ± 35.73 | 25.82 ± 1.19 | 75.27 ± 4.18 | 0.83 ± 0.14 |
| 09:00 | 435.96 ± 95.39 | 29.04 ± 1.61 | 74.07 ± 4.54 | 1.06 ± 0.25 |
| 10:00 | 519.29 ± 90.55 | 31.68 ± 1.99 | 65.95 ± 4.67 | 1.62 ± 0.37 |
| 11:00 | 597.16 ± 141.25 | 33.89 ± 2.06 | 58.98 ± 5.58 | 2.22 ± 0.52 |
| 12:00 | 657.98 ± 75.60 | 34.38 ± 2.14 | 55.43 ± 5.22 | 2.45 ± 0.57 |
| Mean | 412.71 ± 231.75 | 29.96 ± 4.03 | 67.87 ± 9.20 | 1.47 ± 0.72 |

Table S3. Mean values ± standard errors of biomass values for dead saplings of *T. vulgaris* in the drought (n = 8).

| Variables | DS (dead plants) |
| --- | --- |
| Height (cm) | 50.59 ± 3.03 |
| Root collar diameter (mm) | 5.30 ± 0.17 |
| Number of leaves | 7.25 ± 0.53 |
| Leaf dry mass (g) | 3.63 ± 0.24 |
| Stem dry mass (g) | 3.02 ± 0.28 |
| Root dry mass (g) | 1.98 ± 0.31 |
| Aboveground biomass (g) | 6.65 ± 0.47 |
| Total dry mass (g) | 8.63 ± 0.68 |
| Root/shoot ratio (g g^-1^) | 0.30 ± 0.04 |
| Leaf mass fraction (g g^-1^) | 0.43 ± 0.01 |
| Stem mass fraction (g g^-1^) | 0.35 ± 0.02 |
| Root mass fraction (g g^-1^) | 0.22 ± 0.02 |

**
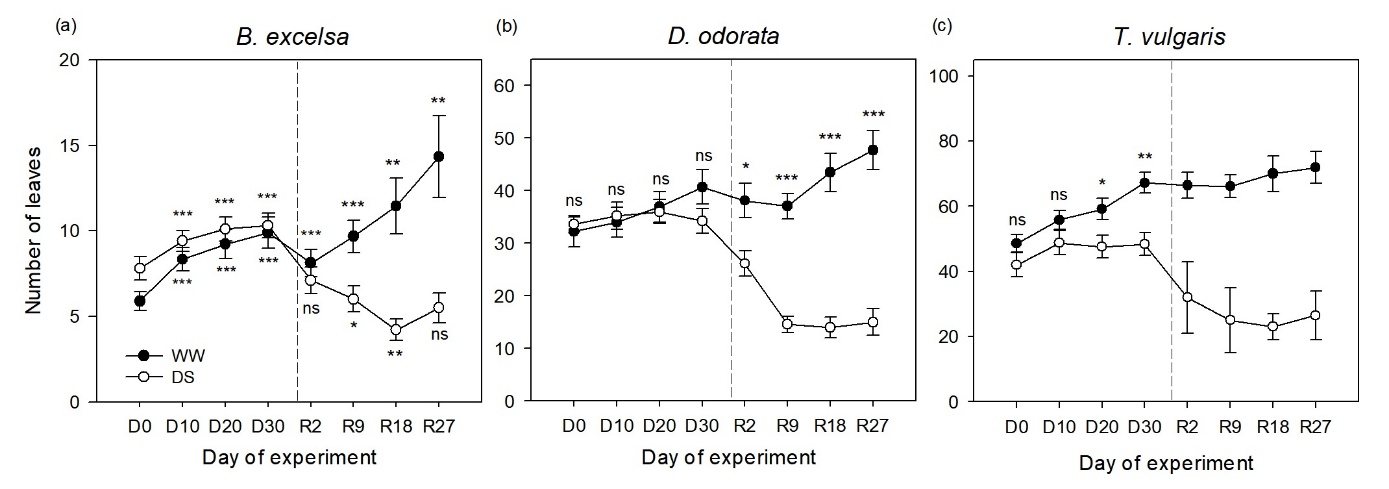
**

Figure S1. Mean values ± standard errors of leaf dynamics of three Amazonian forest species during 62 days of the experiment. WW = well-watered, DS = drought stress. The dashed line represents the last day of irrigation (with a maximum stress of 35 days). Differences between treatments on each day (*D. odorata* and *T. vulgaris*) or over time within each treatment (*B. excelsa*) according to post hoc Tukey are inserted as * *P* < 0.05; ** *P* < 0.01; *** *P* < 0.001; and n.s. (not significant) *P* ≥ 0.05. When comparisons are made over time for the same treatment, the significance of the WW treatment is on the top and that of the DS treatment on the bottom. For *T. vulgaris* in the recovery stage, the values correspond to only surviving saplings (n = 2).


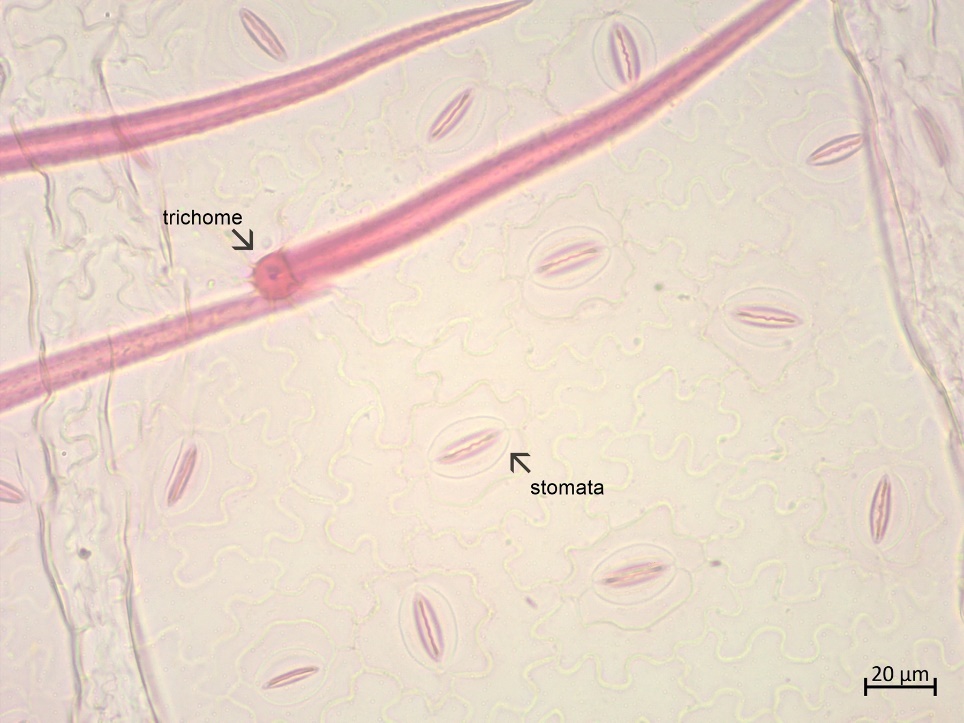


Figure S2. Abaxial side of leaves of T*. vulgaris* where the stomata and tector trichomes are shown.


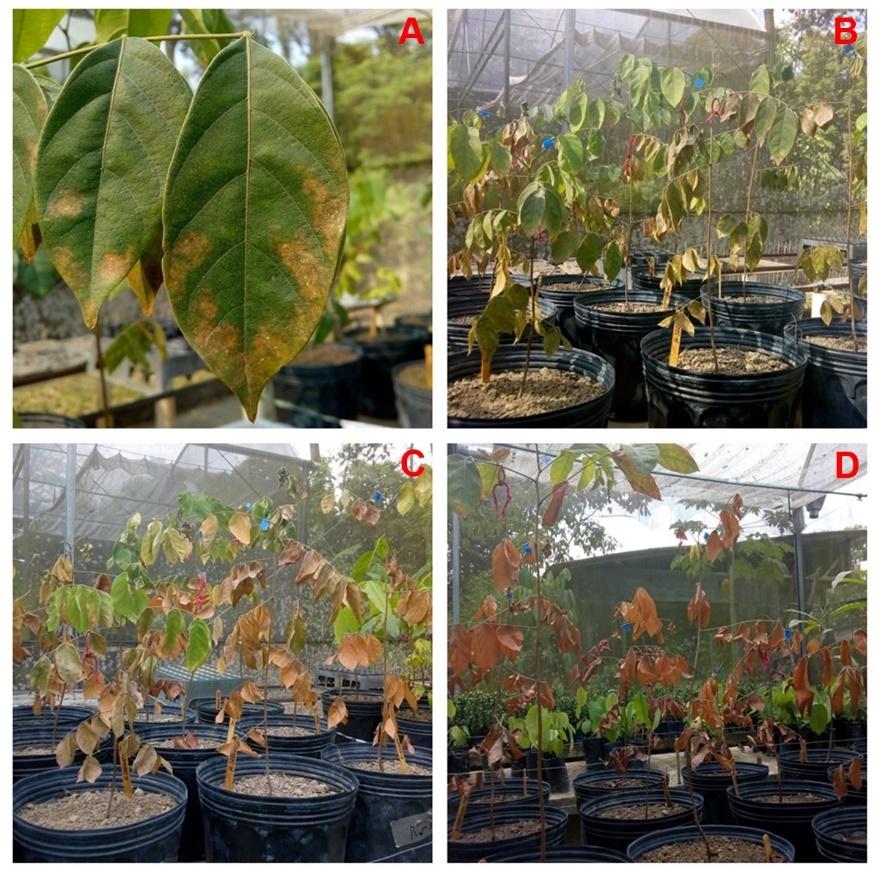


Figure S3. Leaf necrosis in *T. vulgaris* after 20 days (A), 26 days (B) and 35 days (C) of irrigation suspension and after 50 days of the experiment (D).
