# Supplementary material for: Interspecific variation of functional traits in saplings of three Amazonian species under drought stress and recovery
Source: AoB Plants. 2026 Jan 8;18(1):plaf073. doi: 10.1093/aobpla/plaf073 (PMC12833982; doi:10.1093/aobpla/plaf073)
Supplement: plaf073_Supplementary_Data [file plaf073_supplementary_data.zip › Supporting_Information_Figures.pdf]

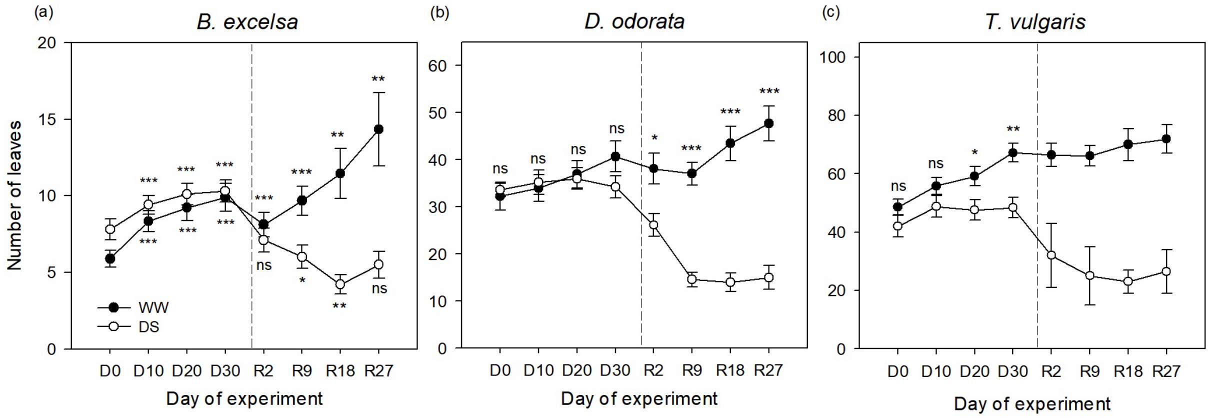

Figure S1. Mean values ± standard errors of leaf dynamics of three Amazonian forest species during 62 days of the experiment. WW = well-watered, DS = drought stress. The dashed line represents the last day of irrigation (with a maximum stress of 35 days). Differences between treatments on each day (*D. odorata* and *T. vulgaris*) or over time within each treatment (*B. excelsa*) according to post hoc Tukey are inserted as * $P < 0.05$; ** $P < 0.01$; *** $P < 0.001$; and n.s. (not significant) $P \geq 0.05$. When comparisons are made over time for the same treatment, the significance of the WW treatment is at the top, and that of the DS treatment on the bottom. For *T. vulgaris* in the recovery stage, the values correspond to only surviving saplings (n = 2).

Alt text. The graph shows the changes in the number of leaves over the days in two phases of the experiment: drought (white circles) and rewatering (black circles) for three native Amazonian species. Statistical differences are also shown.

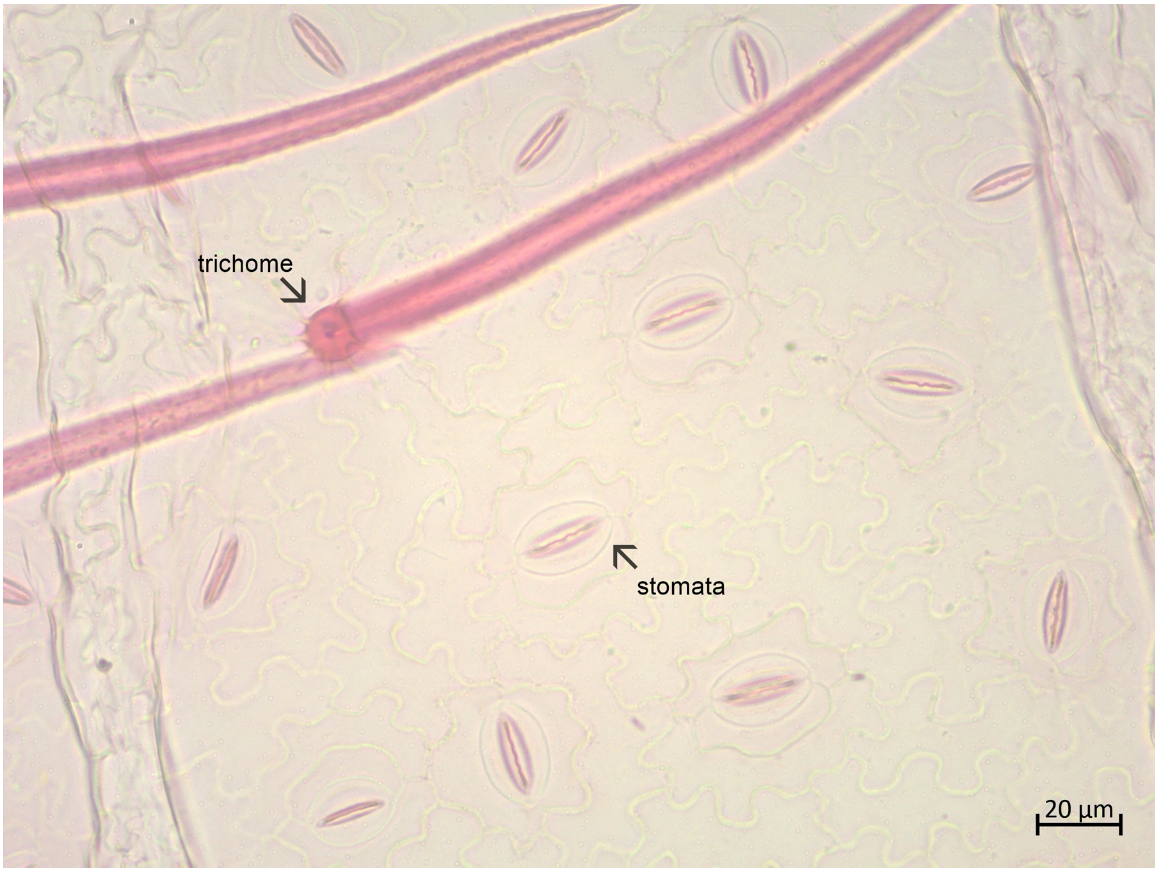

Figure S2. Abaxial side of leaves of *T. vulgaris,* where the stomata and tector trichomes are shown.

Alt text. The figure shows the abaxial side of a leaf of *T. vulgaris,* where stomata and tector trichomes are shown.

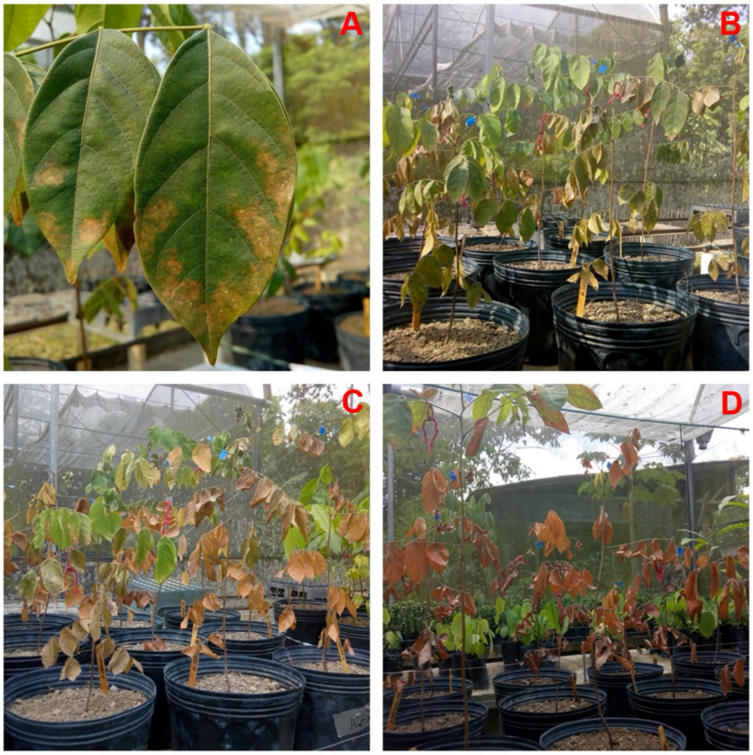

Figure S3. Leaf necrosis in *T. vulgaris* after 20 days (A), 26 days (B), and 35 days (C) of irrigation suspension and after 50 days of the experiment (D).

Alt text. The figure shows the progression of oxidative stress (leaf burn) in *T. vulgaris* during the experiment.
